# Research progress on the role of MZB1 in immune regulation

**DOI:** 10.3389/fimmu.2026.1741773

**Published:** 2026-01-19

**Authors:** Weiqi Liu, Kui Jiang, Minli Jin, Xiaochi Chen

**Affiliations:** 1 Department of The First Affiliated Hospital, Dalian Medical University, Dalian, Liaoning, China; 2 Department of Immunology, College of Basic Medical Science of Dalian Medical University, Dalian, Liaoning, China; 3 Department of Urology, The First Affiliated Hospital of Dalian Medical University, Dalian, China

**Keywords:** antibody production, cell differentiation, immune regulation, MZB1, tumor microenvironment

## Abstract

MZB1 (Marginal Zone B and B1 Cell-Specific Protein), an endoplasmic reticulum-resident protein, has recently garnered significant attention for its role in immune regulation. As a critical modulator within various immune cells, MZB1 influences key processes such as cell differentiation, antibody production, and immune microenvironment dynamics. Despite advances in understanding, the precise mechanisms by which MZB1 contributes to immune homeostasis and dysregulation in pathological conditions remain incompletely defined. Emerging evidence highlights its involvement in tumor biology, inflammatory responses, and autoimmune diseases, positioning MZB1 as a potential therapeutic target. This review aims to synthesize current research findings on MZB1’s functions across different immune cell types, elucidate its interactions with other immune regulatory factors, and explore its impact on the tumor microenvironment and immune-mediated disorders.

## 1 Introduction

The MZB1 has recently been recognized as a multifunctional molecule involved in immune regulation and various pathological conditions (1). MZB1 is predominantly expressed in specialized B cell subsets, including plasma cells, marginal zone B cells and B1 cells, and localizes within the endoplasmic reticulum (ER) (2). Its biological functions encompass acting as a co-chaperone that facilitates integrin activation, antibody folding, and secretion, thereby contributing to the maturation and function of immunoglobulins, particularly IgM and IgA (3). Furthermore, MZB1 regulates calcium flux between the ER and the cytoplasm, which is conducive to proper protein folding. The discovery of multiple isoforms of MZB1, each with distinct functional roles, highlights the complexity of its involvement in immune cell biology and underscores the necessity for further mechanistic investigations (1). Structurally, MZB1 belongs to the CNPY family of ER-resident saposin-like proteins, with recent high-resolution crystallographic studies revealing unique saposin-fold features that may underpin its specialized functions in Ig and integrin maturation (4). These foundational insights into MZB1’s molecular characteristics set the stage for understanding its broader immunological implications.

MZB1’s importance within the immune system is underscored by its pivotal role in B cell differentiation and antibody production. It is preferentially expressed in plasma cells and marginal zone and B1 B cells, subsets that are essential for rapid immune responses to pathogens (3). Functional studies demonstrate that MZB1 promotes the secretion of IgM and IgA by interacting with immunoglobulin heavy chains, a process further modulated by post-translational modifications such as citrullination by peptidylarginine deiminase 2 (PAD2) (3). This modification enhances MZB1’s interaction with IgM/IgA, facilitating efficient antibody secretion, which is crucial for mucosal immunity and systemic defense. Dysregulation of MZB1 expression or function has been implicated in various immune-related diseases, including autoimmune disorders like rheumatoid arthritis and systemic sclerosis, as well as in inflammatory conditions such as periodontitis, where altered MZB1 expression correlates with disease severity and immune cell infiltration (3, 5, 6). Beyond immunity, MZB1 has been associated with the modulation of mitochondrial function and apoptosis in immune and non-immune cells, contributing to cellular homeostasis under stress conditions (7, 8). Collectively, these findings highlight MZB1 as a critical regulator at the intersection of immune cell function, stress responses, and inflammation.

Given its multifaceted roles, studying MZB1 is imperative for advancing our understanding of immune regulation and its pathological perturbations. The protein’s involvement in diverse diseases, ranging from hematological malignancies such as multiple myeloma to solid tumors including ovarian, positions MZB1 as a promising biomarker and therapeutic target (1, 9, 10). For instance, elevated MZB1 expression correlates with disease progression and poor prognosis in multiple myeloma, while in ovarian cancer, higher MZB1 levels are associated with enhanced immune cell infiltration and better clinical outcomes (1, 10). Furthermore, MZB1 influences mitochondrial function and apoptosis pathways via signaling cascades such as AMPK/SIRT1 and PI3K-Akt, implicating it in cardiovascular diseases like myocardial infarction and atherosclerosis, where it confers protective effects by ameliorating mitochondrial dysfunction and inflammation (7, 8). The therapeutic potential of modulating MZB1 is further exemplified by studies showing that compounds like puerarin can upregulate MZB1 expression, thereby attenuating ischemic cardiac injury (11). Additionally, the identification of MZB1 as a key gene in immune-related transcriptomic signatures predictive of disease severity in acute pancreatitis and dengue infection underscores its utility in precision medicine (12, 13). These diverse clinical associations underscore the necessity of continued research into MZB1’s molecular mechanisms and its exploitation as a diagnostic and therapeutic tool across immunological and non-immunological diseases.

## 2 Expression and function of MZB1 in immune cells

### 2.1 MZB1 in B cells

MZB1 is a critical ER-resident protein predominantly expressed in plasma cells, marginal zone (MZ) B cells, and B1 B cells, playing an essential role in B cell differentiation and function. Its expression is markedly elevated in mature and plasma B cells, particularly within specialized B cell subsets such as MZ B cells, which are crucial for rapid antibody responses to blood-borne pathogens. Single-cell transcriptomic analyses in pathological conditions such as chronic rhinosinusitis with nasal polyps (CRSwNP) have demonstrated that MZB1 expression is significantly upregulated in type 2 inflammatory states and is closely associated with the expansion of MZ B cells and plasma cells in affected tissues (1, 14). This suggests that MZB1 is involved in the local differentiation and maturation of B cells into plasma cells. Furthermore, MZB1 has been implicated in the unfolded protein response (UPR) within B cells, facilitating ER expansion and protein folding capacity necessary for high-rate immunoglobulin synthesis. In plasmacytoid dendritic cells (pDCs), a related immune cell type, MZB1 supports ER homeostasis and efficient interferon-α (IFNα) secretion through ATF6-mediated UPR signaling, indicating a broader role in immune cell differentiation and function (15). The regulation of MZB1 itself may be influenced by post-translational modifications such as citrullination by PAD2, which modulates its function during plasmablast differentiation and immunoglobulin secretion (3). A study reveals that miR-185-5p directly targets MZB1, as confirmed by luciferase reporter assays showing binding to a conserved site in the MZB1 3’-UTR. Functionally, overexpression of miR-185-5p significantly suppresses MZB1 expression, while its inhibition elevates MZB1 levels, indicating a robust regulatory relationship. Although direct transcriptional regulators of MZB1 in immunity remain less defined, its dysregulation in autoimmune and inflammatory conditions suggests context-specific upstream control (42). Collectively, these findings underscore MZB1 as a pivotal molecular chaperone that supports B cell differentiation by maintaining ER function and facilitating the transition from mature B cells to antibody-producing plasma cells, particularly under conditions requiring robust humoral responses.

**Figure 1:**
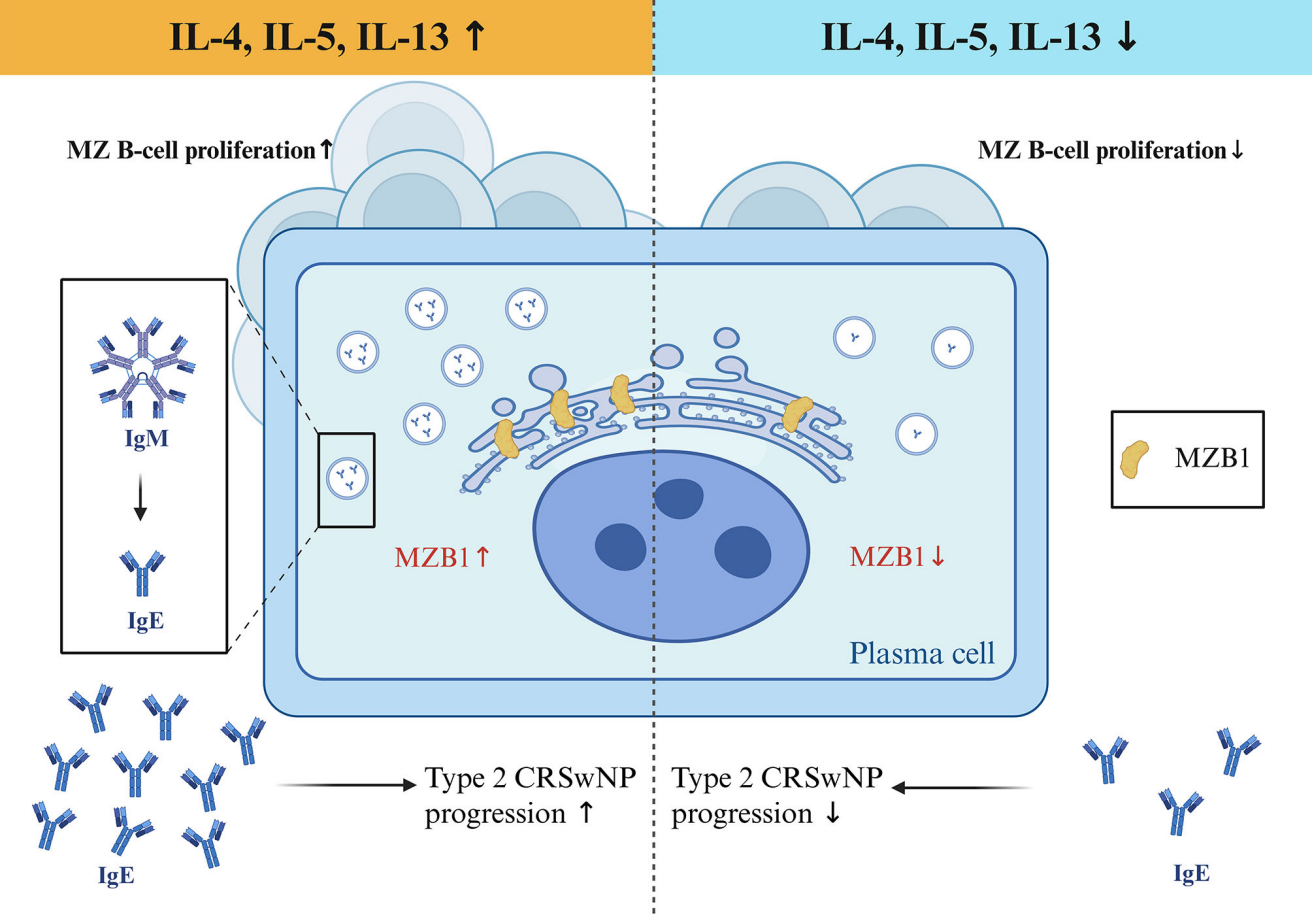
Proposed mechanism of MZB1-mediated immunoregulation in type 2 CRSwNP. This schematic model depicts the central role of the B cell endoplasmic reticulum protein MZB1 in the pathogenesis of Type 2 CRSwNP, contrasting the disease-promoting effects of its upregulation (Left Path) with the therapeutic potential of its suppression (Right Path). In the Type 2 inflammatory milieu (elevated IL-4, IL-5, IL-13), MZB1 expression is upregulated in nasal mucosal B-lineage cells, particularly in plasma cells and MZ B cells. This heightened MZB1 expression facilitates increased local production of immunoglobulin, notably polyclonal IgE, and supports the expansion of the MZ B cell compartment. These events collectively drive the exacerbation of inflammation and promote the development and persistence of nasal polyps. Conversely, targeted downregulation of MZB1 disrupts this pathogenic axis. Reduced MZB1 function leads to decreased local IgE synthesis and a contraction of the disease-associated MZ B cell population. This attenuation of the humoral immune response alleviates Type 2 inflammation and inhibits nasal polyp growth.

MZB1 plays a fundamental role in the generation and secretion of immunoglobulins, particularly IgM and IgA, by acting as a molecular chaperone that assists in the proper folding and assembly of immunoglobulin heavy chains within the ER. Experimental evidence from human primary B cells reveals that MZB1 interacts with the tail pieces of IgM and IgA heavy chains, promoting their secretion. This process is enhanced by PAD2-mediated citrullination of MZB1, which facilitates its interaction with immunoglobulin molecules and supports efficient antibody secretion (3). In pathological contexts such as type 2 CRSwNP, increased MZB1 expression correlates with local IgE overproduction, suggesting that MZB1 not only supports IgM and IgA secretion but may also influence IgE synthesis in mucosal tissues (14, 16)(**Figure 1**). Mechanistically, MZB1 contributes to ER stress responses in B cells, which are tightly linked to immunoglobulin production capacity. Elevated ER stress markers such as HSPA5 and HSP90B1 co-localize with MZB1 in plasma and mature B cells, indicating that MZB1 is integral to managing the high secretory demand during antibody production (16). Moreover, MZB1 influences the formation of polymeric IgA and IgM by promoting the binding of the joining chain (J chain) to immunoglobulins, which is essential for their transcytosis across epithelial barriers and mucosal immunity (17). Clinical and experimental data also link MZB1 expression to disease states characterized by aberrant antibody production, such as certain cancers, where MZB1 levels correlate with prognosis and immune cell infiltration (9, 18, 19). In summary, MZB1 is a critical regulator of antibody biosynthesis and secretion, supporting humoral immunity by ensuring proper immunoglobulin folding, assembly, and ER homeostasis, thereby facilitating effective B cell-mediated immune responses.

### 2.2 MZB1 in dendritic cell function

#### 2.2.1 MZB1’s impact on interferon10.2 secretion

MZB1, a protein resident in the ER, has been identified as a critical regulator of immune cell function beyond its classical role in B cells, notably in pDCs (15). pDCs are specialized for producing large amounts of type I interferons (IFNs), especially IFNα, in response to viral infections and Toll-like receptor (TLR) stimulation (20, 21). Recent studies have demonstrated that MZB1 is highly expressed in pDCs and is essential for efficient IFNα secretion upon TLR9 activation. Mzb1-deficient pDCs exhibit a significant defect in IFNα secretion, which correlates with their impaired ability to promote B cell differentiation into plasma cells, indicating a pivotal role for MZB1 in bridging innate and adaptive immunity. Mechanistically, MZB1 facilitates the expansion of the ER in pDCs following TLR9 stimulation, a process necessary for the high secretory demand of IFNα production. This ER expansion is regulated by the UPR pathway, particularly through activating transcription factor 6 (ATF6). Mzb1 knockout pDCs show defective ATF6 activation, and pharmacological inhibition of ATF6 cleavage in wild-type pDCs recapitulates the IFNα secretion defects observed in Mzb1-deficient cells. Therefore, MZB1 supports IFNα secretion by mitigating ER stress and promoting the ATF6-mediated UPR, which is critical for the secretory function of pDCs during immune responses. This functional role highlights MZB1 as a key modulator of innate antiviral immunity and suggests that its dysregulation could impair host defense mechanisms that depend on robust type I IFN responses (15).

#### 2.2.2 The relationship between MZB1 and endoplasmic reticulum stress response

The connection between MZB1 and ER stress response is central to understanding its function in dendritic cells. The ER is responsible for the folding and processing of secretory and membrane proteins, and its capacity is challenged during immune activation when cells increase protein synthesis. This can lead to ER stress, triggering the UPR to restore homeostasis. MZB1, as an ER-resident protein, is implicated in modulating this stress response, particularly through the ATF6 branch of the UPR. In pDCs stimulated via TLR9, MZB1 facilitates the expansion of the ER and proper activation of ATF6, which in turn upregulates genes that enhance protein folding and secretion capacity. The absence of MZB1 results in a failure to adequately activate ATF6, leading to unresolved ER stress and diminished secretion of IFNα. This suggests that MZB1 acts as a chaperone or co-chaperone that supports the folding and assembly of proteins critical for immune signaling and secretion. Beyond pDCs, the role of MZB1 in ER stress response may extend to other immune cells, as its expression correlates with immune activation states and is associated with immune-related diseases. For example, increased MZB1 expression has been observed in pathological conditions characterized by immune dysregulation, such as autoimmune diseases and certain cancers, where ER stress and UPR pathways are often perturbed. The ability of MZB1 to modulate ER stress responses and maintain cellular secretory function underscores its importance in immune homeostasis and suggests potential therapeutic targeting in diseases where ER stress contributes to immune dysfunction (15, 22).

## 3 Interaction between MZB1 and tumor microenvironment

### 3.1 MZB1 in ovarian cancer

#### 3.1.1 The relationship between MZB1 and endoplasmic reticulum stress response

MZB1 has emerged as a significant biomarker in ovarian cancer, with study underscoring its prognostic relevance. Analysis of 381 ovarian cancer samples from the TCGA database revealed that elevated MZB1 expression correlates positively with improved clinical outcomes, suggesting that MZB1 may serve as a favorable prognostic marker in this malignancy (10). This association was further supported by survival analyses that identified MZB1 among four core immune-related genes—alongside CXCL9, CD79A, and MS4A1—significantly linked to both tumor-infiltrating immune cells (TICs) and patient survival in ovarian cancer (23). Proteomic profiling of serous ovarian tumors also demonstrated differential expression of MZB1, with notably higher levels in low-grade serous cystadenocarcinoma compared to borderline tumors, indicating its potential role in tumor progression and classification (24). Moreover, MZB1 was incorporated into a novel chemotherapy benefit index (CBI) developed through Bayesian network and machine learning analyses, where its expression contributed to stratifying patients by prognosis and predicting response to chemotherapy and immunotherapy (25). Collectively, these findings position MZB1 as a promising biomarker that not only reflects tumor biology but also aids in prognostication and therapeutic decision-making in ovarian cancer.

#### 3.1.2 Regulatory role of MZB1 in the tumor immune microenvironment

Beyond its prognostic value, MZB1 plays a crucial role in modulating the TIME of ovarian cancer. High MZB1 expression is associated with increased infiltration of immune cells within the tumor milieu, suggesting that MZB1 may enhance anti-tumor immunity by fostering a more immunologically active microenvironment. Functional assays demonstrated that elevated MZB1 levels inhibit the migration and proliferation of ovarian cancer SKOV3 cells, indicating a direct suppressive effect on tumor aggressiveness potentially mediated through immune-related pathways (10). Advanced spatial transcriptomics analyses further elucidated MZB1’s spatial distribution, revealing its enrichment in B cell-dense regions within ovarian tumors, which underscores its involvement in shaping localized immune responses (26). The integration of MZB1 into immune-related gene signatures and its correlation with TICs reinforce its role as a modulator of immune cell dynamics, likely influencing the balance between tumor-promoting and tumor-suppressing immune elements (23). Furthermore, molecular characterization of patient subgroups stratified by chemotherapy benefit index revealed that MZB1 expression aligns with immune activation signatures and predicts better responsiveness to immune checkpoint blockade therapies, highlighting its potential as a therapeutic target to enhance immunotherapy efficacy in ovarian cancer (25). Taken together, these data suggest that MZB1 is not only a biomarker but also an active participant in the immunoregulatory networks within the ovarian cancer microenvironment, offering avenues for novel immunomodulatory interventions.

### 3.2 MZB1 in other cancers

**Figure 2:**
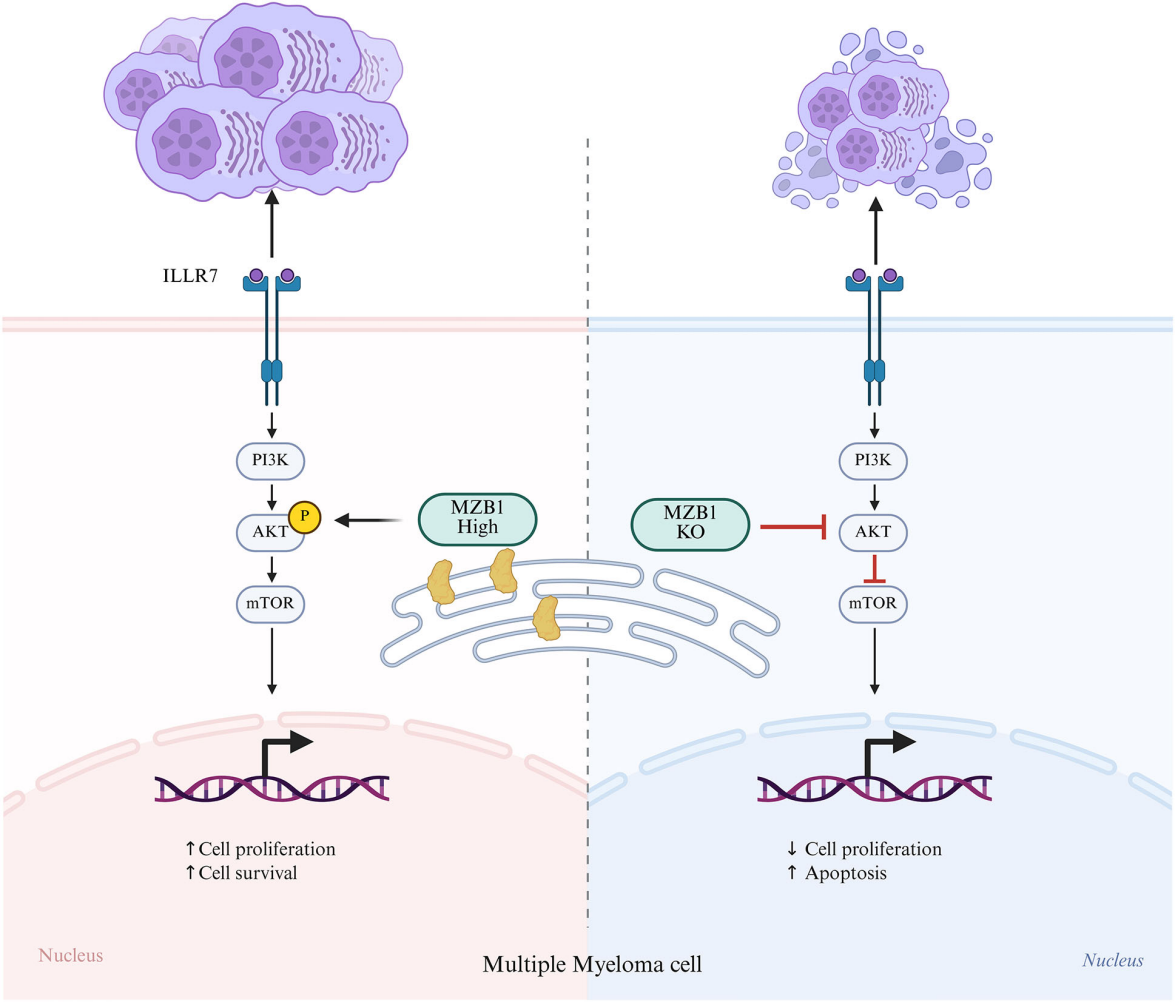
MZB1 modulates MM cell proliferation through regulation of the AKT pathway. The schematic illustrates the proposed mechanism by which MZB1 expression influences multiple myeloma (MM) pathogenesis. (Left Panel: MZB1 Upregulation) Elevated MZB1 expression promotes activation of the PI3K-AKT-mTOR axis, leading to phosphorylation of downstream targets. (Right Panel: MZB1 Knockdown) Genetic silencing of MZB1 expression significantly inhibits PI3K-AKT-mTOR pathway activation, reduces expression of proliferation markers, and substantially impairs MM cell growth and clonogenic potential.

MZB1 has garnered increasing attention for its multifaceted roles in various cancers beyond its classical functions in immune regulation. In hematologic malignancies such as multiple myeloma (MM) and chronic lymphocytic leukemia (CLL), MZB1 has emerged as a significant molecular player influencing disease progression, prognosis, and potentially therapeutic targeting. Proteomic and functional studies have demonstrated that MZB1 is upregulated in MM patient-derived mononuclear cells, where its elevated expression correlates with enhanced malignant progression. Specifically, label-free quantitative proteomics identified MZB1 among 192 differentially regulated proteins in MM cells, with functional assays in RPMI-8226 MM cell lines confirming that higher MZB1 expression promotes MM pathogenesis, suggesting its candidacy as a novel biomarker and therapeutic target in MM (9)(**Figure 2**). Moreover, alternative RNA splicing analyses in MM revealed that MZB1 is among the differentially spliced genes associated with SF3B1 mutations, which are linked to aberrant splicing patterns and adverse survival outcomes. This highlights a potential role for MZB1 not only at the expression level but also in post-transcriptional regulation contributing to MM heterogeneity and disease aggressiveness (27).

In CLL, elevated MZB1 expression has also been correlated with poor prognosis. A comprehensive review underscored MZB1’s role as a proapoptotic caspase-binding protein and co-chaperone involved in integrin activation and antibody secretion, with dysregulation implicated in cancer progression, including CLL. High MZB1 levels in CLL patients are associated with unfavorable clinical outcomes, further supporting its utility as a prognostic marker and potential immunomodulatory target (1). The mechanistic underpinnings involve MZB1’s regulation of calcium flux between the ER and cytoplasm, maintaining ER homeostasis crucial for B-cell function and survival. Dysregulated MZB1 expression in malignant B cells may thus contribute to tumor cell survival and proliferation by modulating ER stress responses and apoptotic pathways. What’s more, MZB1 expression was significantly higher in patients with unmutated IGHV status (39). As emphasized in the recent systematic review by Maher et al., epigenetic regulatory mechanisms are key determinants of heterogeneity, immune evasion, and therapy resistance in B-cell neoplasms, supporting the importance of studying these alterations. Therefore, investigating the regulation of MZB1 expression should be approached within a defined epigenetic context, including DNA methylation, histone modifications, and non-coding RNA regulation. Its overexpression could be mediated by mechanisms such as promoter hypomethylation, enrichment of activating histone marks at enhancers, or inactivation of specific miRNAs (40).

Beyond hematologic malignancies, pan-cancer analyses have revealed that MZB1 is significantly overexpressed in multiple cancers, including acute myeloid leukemia, and its expression correlates with tumor stage, immune checkpoint gene expression, tumor microenvironment (TME) infiltration, and patient survival. In myeloid leukemia cell lines, MZB1 knockdown suppresses proliferation, emphasizing its functional relevance in leukemic cell growth (19). These findings collectively suggest that MZB1 serves as a critical nexus linking ER stress, immune regulation, and tumor biology in hematologic cancers.

Recent bioinformatics and functional studies have specifically implicated MZB1 in shaping the tumor-immune microenvironment of melanoma; moreover, research has demonstrated that MZB1 is overexpressed in human melanoma cell lines. Building upon its established immunoregulatory functions in other cancer contexts, we investigated its tumor cell–intrinsic role. The knockdown of MZB1 in melanoma cells augments the expression of multiple genes involved in promoting anti-tumor immune responses. This suggests that elevated MZB1 expression may contribute to an immunosuppressive environment, and its inhibition could potentially enhance tumor immunogenicity (41).

In summary, the accumulated evidence positions MZB1 as a pivotal molecule in the pathogenesis and progression of MM, CLL and melanoma. Its overexpression and functional involvement in malignant plasma and B cells underscore its potential as a prognostic biomarker and therapeutic target. Future research aimed at elucidating the molecular mechanisms of MZB1, including its isoform-specific functions and interactions within the ER stress and immune signaling pathways, may provide novel avenues for targeted interventions in these cancers.

## 4 The relationship between MZB1 and autoimmune diseases

### 4.1 MZB1 in rheumatoid arthritis

#### 4.1.1 MZB1 citrullination and its impact on B cell differentiation

Recent research has elucidated that MZB1 undergoes citrullination, a post-translational modification catalyzed by peptidylarginine deiminases (PADs), especially PAD2, which converts peptidylarginine to peptidylcitrulline. This modification has been shown to be enriched in rheumatoid arthritis-associated interstitial lung disease (RA-ILD), implicating a disease-specific role of MZB1 citrullination in RA pathogenesis (3). The citrullination of MZB1 occurs during the differentiation of human plasmablasts and is essential for promoting the secretion of IgM and IgA antibodies. Pharmacological inhibition or genetic ablation of PAD2 diminishes MZB1 citrullination, which in turn attenuates IgM and IgA secretion without affecting IgG secretion or the differentiation of plasmablasts expressing these immunoglobulins. This suggests that citrullinated MZB1 specifically modulates the secretory function of B cells rather than their differentiation per se. Furthermore, the interaction between MZB1 and IgM/IgA heavy chains is disrupted upon PAD2 inhibition, indicating that citrullination facilitates the physical association necessary for efficient antibody secretion. These findings highlight a novel regulatory mechanism whereby PAD2-mediated citrullination of MZB1 enhances immunoglobulin secretion, contributing to the aberrant humoral immune responses characteristic of RA. Given the centrality of autoantibody production in RA pathogenesis, particularly anti-citrullinated protein antibodies (ACPAs), the citrullination of MZB1 represents a critical molecular event linking B cell function to autoimmune inflammation (3). This mechanistic insight opens potential therapeutic avenues targeting PAD2 or MZB1 citrullination to modulate pathogenic antibody production in RA.

#### 4.1.2 MZB1 and its relationship with inflammatory responses

Beyond its role in B cell differentiation and antibody secretion, MZB1 has emerged as a significant player in the inflammatory milieu of rheumatoid arthritis. Transcriptomic analyses of whole blood from RA patients undergoing tumor necrosis factor inhibitor (TNFi) therapy identified MZB1 as a biomarker associated with treatment response, underscoring its involvement in disease activity and immune regulation (28). MZB1’s expression correlates with B cell development and antibody production, which are pivotal in driving the autoimmune process and formation of antidrug antibodies that can impair therapeutic efficacy. Moreover, studies targeting MyD88, a key adaptor in innate immune signaling pathways, demonstrated that inhibition of MyD88 downregulated MZB1 expression alongside proinflammatory cytokines and chemokines in peripheral blood mononuclear cells (PBMCs) from DMARD-naïve RA patients (29). This downregulation coincided with attenuation of B cell receptor signaling pathways, implicating MZB1 in the amplification of inflammatory cascades. Additionally, MZB1 was identified among a set of genes involved in pathogenic processes within the RA synovium, suggesting its functional relevance in local joint inflammation and tissue damage (29). The presence of MZB1-expressing plasma cells in the bone marrow of RA patients further supports its role in sustaining chronic autoimmunity, as these cells produce disease-associated autoantibodies, including ACPAs that target citrullinated proteins and activate neutrophils, thereby perpetuating inflammation (30). Bioinformatics analyses comparing RA with spondyloarthritis also revealed that MZB1 expression is closely linked with immune cell subsets such as T cells, NK cells, and dendritic cells, indicating a broader immunomodulatory role beyond B cells and potential utility as a diagnostic marker distinguishing autoimmune conditions (22). Collectively, these data position MZB1 as a nexus between adaptive immune dysregulation and inflammatory signaling in RA, contributing to disease progression and therapeutic resistance. Targeting MZB1 or its regulatory pathways may thus represent a promising strategy to mitigate inflammation and improve clinical outcomes in RA patients.

### 4.2 MZB1 in other autoimmune diseases

MZB1 has garnered attention for its multifaceted roles in immune regulation, particularly in autoimmune diseases such as systemic lupus erythematosus (SLE). SLE is a clinically heterogeneous autoimmune disorder characterized by diverse clinical manifestations and serological abnormalities. Recent proteomic analyses have identified MZB1 as a protein of interest associated with disease activity and organ involvement in SLE. In a large cohort study involving 223 patients with active SLE, unsupervised clustering of plasma proteomes revealed distinct patient subgroups with differential clinical and serological features. Notably, one cluster (C6) associated with renal involvement showed increased expression of MZB1 alongside other proteins such as SND1 and AGL (31).

Moreover, MZB1’s functional impact extends to modulating intracellular calcium flux between the ER and cytoplasm, maintaining an oxidizing environment necessary for proper protein folding. Disruption of calcium homeostasis by MZB1 depletion can activate downstream signaling pathways implicated in immune activation and inflammation, processes central to SLE pathophysiology (1). Given that SLE involves complex immune dysregulation, including aberrant B cell receptor signaling and cytokine production, MZB1’s regulatory role in these pathways may contribute to disease severity and organ damage.

Beyond its biological functions, MZB1 has been proposed as a therapeutic target. Its expression correlates with disease activity and organ involvement, suggesting that modulating MZB1 or its downstream effects could ameliorate pathological B cell responses. This is supported by findings in related autoimmune diseases such as rheumatoid arthritis, where targeting upstream signaling molecules affects MZB1 expression and inflammatory mediators (29). Although direct interventions targeting MZB1 in SLE remain to be developed, the protein’s involvement in antibody secretion and immune cell differentiation positions it as a promising candidate for future therapeutic strategies.

In summary, emerging evidence highlights MZB1 as an important player in the immunopathogenesis of SLE. Its elevated expression in patient subgroups with active disease and renal involvement, coupled with its critical roles in B cell function and calcium homeostasis, underscores its potential as both a biomarker and a therapeutic target. Further research is warranted to elucidate the precise molecular mechanisms by which MZB1 contributes to SLE heterogeneity and to explore targeted interventions that may improve patient outcomes.

## 5 Molecular mechanisms and signaling pathways of MZB1

### 5.1 MZB1 and its interaction with calcium signaling pathways

#### 5.1.1 MZB1’s role in maintaining endoplasmic reticulum calcium homeostasis

MZB1 has been increasingly recognized as a critical regulator of calcium homeostasis within the ER, a key organelle responsible for calcium storage and signaling in immune cells (4). MZB1 functions as a co-chaperone that modulates calcium flux between the ER and cytoplasm, thereby maintaining the oxidizing environment essential for proper protein folding and cellular homeostasis. Specifically, MZB1 helps preserve calcium levels inside the ER, which is crucial for sustaining the ER’s functional integrity. Loss or depletion of MZB1 disrupts this delicate balance, leading to elevated cytosolic calcium concentrations and subsequent activation of downstream signaling cascades. This disruption not only affects calcium-dependent enzymatic activities but also impairs the ER’s capacity to manage oxidative stress, which can trigger apoptotic pathways (2, 4). The existence of multiple MZB1 isoforms with distinct functions further complicates its regulatory role in calcium homeostasis, suggesting that each isoform may fine-tune calcium signaling in specific immune contexts or cell types. Thus, MZB1 acts as a pivotal molecular hub that integrates calcium homeostasis with protein folding and immune cell function, underscoring its importance in maintaining cellular equilibrium and preventing pathological states linked to calcium dysregulation (1).

#### 5.1.2 Impact of calcium signaling pathways on immune cell function

Calcium signaling is fundamental to the regulation of immune cell activities, influencing processes such as activation, differentiation, cytokine production, and apoptosis (32, 33). The role of MZB1 in modulating ER calcium stores directly impacts these calcium-dependent pathways, thereby shaping immune responses. Elevated cytosolic calcium levels, resulting from impaired MZB1 function, can activate a variety of calcium-sensitive signaling molecules and transcription factors that drive immune cell proliferation and effector functions (34). Moreover, calcium signaling is essential for antibody folding and secretion, processes that are critically dependent on ER function and are regulated by MZB1. Dysregulation of calcium homeostasis due to altered MZB1 expression has been linked to immune dysfunction and pathological conditions such as inflammation and autoimmunity. The interplay between MZB1 and calcium signaling pathways thus represents a key axis by which immune cells maintain their functional competence and respond appropriately to environmental cues (1). Understanding this interaction provides valuable insights into the molecular mechanisms underlying immune regulation and highlights potential therapeutic targets for diseases characterized by immune dysregulation.

### 5.2 Isoforms of MZB1 and their functional diversity

#### 5.2.1 MZB1 isoforms’ biological functions

One of the key revelations about MZB1 is the existence of five distinct isoforms, each contributing uniquely to its biological roles. These isoforms function primarily as co-chaperones that facilitate integrin activation, antibody folding, and secretion, processes essential for effective immune responses. Furthermore, MZB1 isoforms are involved in maintaining calcium homeostasis by regulating calcium flux between the ER and cytoplasm, preserving an oxidizing environment within the ER crucial for proper protein folding. Disruption of MZB1 expression leads to increased cytosolic calcium levels and activation of downstream signaling pathways, highlighting its role in cellular signaling and homeostasis. The diversity of MZB1 isoforms allows fine-tuning of these processes, enabling immune cells to adapt to varying physiological demands. This isoform-specific functionality underscores the complexity of MZB1’s involvement in immune cell differentiation and function, suggesting that each isoform may have specialized roles in modulating immune responses and maintaining cellular integrity (1).

#### 5.2.2 Specific roles of isoforms in disease

The functional heterogeneity of MZB1 isoforms extends into their involvement in pathological conditions. Dysregulation of MZB1 expression and isoform balance has been implicated in a spectrum of diseases, including cancers such as multiple myeloma, CLL, and diffuse large B-cell lymphoma (DLBCL). Elevated MZB1 levels correlate with disease progression and poor prognosis in these malignancies, indicating that certain isoforms may contribute to tumorigenesis or tumor maintenance by affecting plasma cell function and survival. Beyond oncology, MZB1 isoforms have been linked to immune-mediated diseases and inflammatory conditions. For example, single-cell transcriptomic analyses have identified expansions of MZB1-expressing plasma cells in acute pancreatitis, associating these cells with disease severity and recovery, which suggests isoform-specific roles in modulating immune responses during inflammation. Additionally, spatial transcriptomic studies have revealed MZB1 as a potential therapeutic target in ovarian cancer, emphasizing the clinical relevance of its isoforms in disease microenvironments. The presence of distinct isoforms likely enables MZB1 to participate in diverse cellular pathways that contribute to disease pathogenesis, making it a promising candidate for targeted diagnostic and therapeutic strategies. Continued research into the molecular mechanisms and isoform-specific functions of MZB1 is essential for understanding its multifaceted roles in health and disease (1, 13, 26).

## 6 Clinical application prospects of MZB1

### 6.1 MZB1 as a potential biomarker

#### 6.1.1 MZB1 in cancer prognosis

MZB1 has emerged as a promising biomarker in various cancers, reflecting its involvement in tumor progression, immune modulation, and patient prognosis. Pan-cancer analyses reveal that MZB1 is significantly overexpressed in multiple tumor types including kidney renal clear cell carcinoma (KIRC), breast invasive carcinoma, and acute myeloid leukemia, where its expression correlates with clinical stage, overall survival, and progression-free survival, underscoring its prognostic relevance (19). In breast cancer specifically, MZB1 expression is predominantly found in estrogen receptor-positive tumors, with higher expression linked to advanced disease stages and poorer disease-free survival, establishing MZB1 as an independent prognostic factor in this subgroup (18). Additionally, proteomic profiling in renal neoplasms identified MZB1 as a diagnostic biomarker capable of clinically distinguish mucinous tubular and spindle cell carcinoma (MTSCC) serving as sensitive and specific markers, with an inverse correlation between MZB1 levels and tumor stage, suggesting a potential anti-tumor role via complement system activation (35). In pancreatic adenocarcinoma, MZB1 is part of a five-gene prognostic signature related to B cell infiltration, with higher expression associated with worse survival outcomes, indicating its utility in risk stratification and highlighting the immune microenvironment’s role in tumor progression (36). Furthermore, in ovarian cancer, MZB1 expression correlates with tumor-infiltrating immune cells and patient survival, suggesting its value as both a prognostic biomarker and a potential immunotherapeutic target (23). Beyond solid tumors, MZB1 expression influences leukemic cell proliferation, as knockdown experiments in myeloid leukemia cell lines demonstrate reduced proliferation, supporting its functional significance in cancer biology (19). **Table 1** summarizes the expression status and prognostic relevance of MZB1 in different cancers. Collectively, these findings underscore MZB1’s multifaceted role in cancer prognosis, reflecting tumor stage, immune infiltration, and patient outcomes, and position it as a candidate biomarker for diagnostic and therapeutic applications across diverse malignancies.

**Table 1:** The expression status and prognostic relevance of MZB1 in different cancers.

Cancer type	Expression status	Prognostic relevance	Role (Promoter/suppressor)	Key immune-related functions
Ovarian Cancer	High expression	Favorable prognosis. Associated with better clinical outcomes.	Tumor suppressor, Inhibits migration and proliferation of cancer cells *in vitro*.	Correlates with increased tumor-infiltrating immune cells (especially B cells) and an immunologically active tumor microenvironment.
Multiple Myeloma	High expression/Upregulated	Poor prognosis. Correlates with disease progression.	Tumor promoter. Contributes to pathogenesis.	Functions are linked to ER stress and immunoregulation in malignant plasma cells (e.g., affecting antibody secretion and survival).
Chronic Lymphocytic Leukemia	High expression	Poor prognosis. Associated with unfavorable outcomes.	Tumor promoter. Linked to disease progression.	Acts in pathways related to integrin activation and antibody secretion.
Acute Myeloid Leukemia	Significantly overexpressed	Correlates with tumor stage and patient survival. Knockdown inhibits proliferation.	Tumor promoter (functionally implied).	Its overexpression is associated with immune checkpoint gene expression and tumor microenvironment infiltration.
Breast Invasive Carcinoma	High expression (Noted in pan-cancer analysis)	Poor prognosis (from pan-cancer data).	Tumor promoter (inferred from association).	It has no clear function
Kidney Renal Clear Cell Carcinoma	Significantly overexpressed (Noted in pan-cancer analysis)	Correlates with clinical stage and survival (from pan-cancer data).	Not specified.	It has no clear function
Pancreatic Adenocarcinoma	High expression (as part of a prognostic signature)	Poor prognosis. High expression correlates with worse survival.	Tumor promoter (inferred from prognostic association).	Its expression is associated with B-cell infiltration in the tumor microenvironment.
Diffuse Large B-cell Lymphoma	Elevated (Context for B-cell lymphomas)	Poor prognosis (context for B-cell lymphomas).	Tumor promoter.	Its function is directly related to antibody secretion and survival pathways in malignant B cells.
Melanoma	High expression	Implied poor prognosis	Tumor promoter	Has immune-related functions. Its mechanisms are linked to remodeling the tumor microenvironment and promoting immune escape

#### 6.1.2 MZB1 in diagnostic value for autoimmune diseases

MZB1 also holds significant diagnostic potential in autoimmune diseases, where its expression reflects immune dysregulation and disease activity. In systemic sclerosis (SSc), elevated serum MZB1 levels are associated with extensive skin fibrosis and pulmonary fibrosis, indicating its utility as a biomarker for disease severity and organ involvement (37). Proteomic analyses in SLE patients reveal distinct proteomic clusters with MZB1 contributing to the classification of active renal disease, suggesting its involvement in disease heterogeneity and potential as a biomarker for organ-specific manifestations (31). In rheumatoid arthritis (RA), transcriptomic studies identify MZB1 as a novel biomarker linked to treatment response to tumor necrosis factor inhibitors, with its expression associated with B cell development and antibody production, implicating it in antidrug antibody formation and therapeutic efficacy (28). Moreover, in periodontitis, an inflammatory autoimmune-related condition, MZB1 is upregulated in gingival tissues, implicating it in B cell-mediated immune responses and highlighting its promise as a biomarker for disease pathogenesis (6). In psoriasis vulgaris patients with reduced high-density lipoprotein levels, MZB1 is among a set of biomarkers identified in both blood and skin samples, linking lipid metabolism alterations to immune dysregulation and disease exacerbation (38). These studies collectively demonstrate that MZB1 serves as a sensitive indicator of immune activation and tissue involvement in autoimmune conditions, offering diagnostic value and potential guidance for personalized treatment strategies. Its consistent association with B cell function and immune response modulation across autoimmune diseases further supports its candidacy as a biomarker for disease diagnosis, activity monitoring, and therapeutic response prediction.

### 6.2 Future research directions and challenges

#### 6.2.1 MZB1 research technical challenges

Research on MZB1 faces several technical challenges that stem from the protein’s complex biology and multifaceted roles in immune regulation. One major hurdle is the presence of five distinct isoforms of MZB1, each exhibiting specific functional roles within cellular processes. This isoform diversity complicates the design of experiments aimed at delineating precise molecular mechanisms, as differential expression patterns and overlapping functions may confound interpretation of results. Additionally, MZB1’s involvement in critical cellular activities such as integrin activation, antibody folding, secretion, and calcium flux regulation between the ER and cytoplasm requires sophisticated methodologies capable of capturing dynamic intracellular events. Techniques such as advanced proteomics, live-cell imaging, and high-resolution structural analysis are essential but often limited by sensitivity and specificity constraints. Furthermore, the regulation of calcium homeostasis by MZB1, which maintains an oxidizing environment in the ER, poses challenges in accurately measuring localized calcium concentrations and redox states in real time. Disruption of MZB1 leads to elevated cytosolic calcium and activation of downstream signaling pathways, highlighting the need for precise tools to dissect these signaling cascades. Another technical barrier is the integration of data from diverse pathological contexts where MZB1 is implicated, including cancer, obesity, inflammation, and autoimmune diseases. The heterogeneity of these conditions demands robust models that can recapitulate disease-specific microenvironments to study MZB1’s role effectively. Lastly, the development of specific antibodies or inhibitors that can selectively target individual MZB1 isoforms remains a significant challenge, limiting the ability to translate mechanistic insights into therapeutic interventions. Addressing these technical challenges will require multidisciplinary approaches combining molecular biology, biochemistry, bioinformatics, and clinical sciences to unravel MZB1’s complex biology comprehensively.

#### 6.2.2 Future clinical trials and application prospects

The future clinical translation of MZB1 research holds promising potential, particularly in the realms of diagnostics and therapeutics for immune-related diseases and cancers. Elevated MZB1 expression has been linked to poor prognosis in CLL and diffuse large B-cell lymphoma (DLBCL), underscoring its utility as a prognostic biomarker. This prognostic potential invites clinical trials aimed at validating MZB1 expression levels as a stratification tool for patient risk assessment and treatment customization. Moreover, the association of MZB1 upregulation with multiple myeloma progression suggests that therapeutic targeting of MZB1 or its downstream pathways could offer novel treatment avenues. Future clinical trials will need to evaluate the safety and efficacy of such targeted therapies, possibly involving small molecules or monoclonal antibodies designed to modulate MZB1 activity or expression. Given MZB1’s role as a co-chaperone in antibody folding and secretion, manipulating its function could also enhance the efficacy of antibody-based immunotherapies. Additionally, since MZB1 regulates calcium homeostasis and ER stress responses, clinical interventions targeting these pathways might ameliorate pathological conditions characterized by MZB1 dysregulation, such as autoimmune diseases and inflammatory disorders. However, the complexity of MZB1 isoforms necessitates that clinical trials incorporate biomarker-driven patient selection and isoform-specific monitoring to optimize therapeutic outcomes. The integration of proteomic and functional analyses into trial designs will be critical for identifying responders and understanding resistance mechanisms. Furthermore, the development of non-invasive diagnostic tools measuring circulating MZB1 or its isoforms could facilitate early detection and monitoring of disease progression. Overall, the clinical application prospects of MZB1 research are promising but require rigorous translational studies and well-designed clinical trials to realize their full potential in personalized medicine.

## 7 Discussion

In conclusion, MZB1 has emerged as a pivotal immunoregulatory factor with multifaceted roles across various immune cell types, underscoring its significance in maintaining immune homeostasis and modulating immune responses. From an expert perspective, the accumulated evidence highlights MZB1’s intricate involvement not only in normal immune function but also in the pathogenesis of complex disorders such as malignancies and autoimmune diseases. This dual role positions MZB1 at a critical nexus where immune regulation intersects with disease development, making it a compelling focus for ongoing and future research.

Balancing the diverse research perspectives, it is clear that while numerous studies have delineated the expression patterns and functional attributes of MZB1 in immune cells—particularly B cells, plasma cells, and subsets of T cells—there remains a gap in fully elucidating the molecular mechanisms by which MZB1 orchestrates immune modulation. Some investigations emphasize its role in protein folding and calcium homeostasis within the endoplasmic reticulum, which are essential for antibody secretion and cellular stress responses. Others suggest that MZB1 influences signaling pathways that govern immune cell activation, differentiation, and survival. Reconciling these findings requires integrative approaches that combine molecular biology, immunology, and systems biology to map the comprehensive network of MZB1 interactions and downstream effects.

Furthermore, the impact of MZB1 in pathological contexts such as tumors and autoimmune diseases is gaining increasing recognition. In oncology, aberrant MZB1 expression has been linked to tumor immune evasion and altered tumor microenvironments, suggesting that MZB1 may serve as both a biomarker and a therapeutic target. MZB1 is not merely a passive biomarker but an active contributor to tumor progression through both cell-autonomous proliferative mechanisms and profound immunomodulatory effects. Conversely, in autoimmune conditions, dysregulated MZB1 function appears to contribute to the breakdown of self-tolerance and chronic inflammation. These insights advocate for a nuanced understanding of MZB1’s context-dependent roles, which is essential for developing targeted interventions that can modulate its activity without compromising physiological immune functions.

Looking ahead, the future of MZB1 research lies in deepening mechanistic insights and translating these findings into clinical applications. Advanced techniques such as single-cell RNA sequencing, CRISPR-based gene editing, and high-resolution imaging will be instrumental in dissecting MZB1’s cell-specific functions and regulatory networks. Additionally, preclinical models that mimic human disease states will be invaluable for testing the therapeutic potential of modulating MZB1 activity. Clinically, the development of MZB1-targeted diagnostics and therapeutics could revolutionize the management of immune-related diseases, offering more precise and effective strategies for diagnosis, prognosis, and treatment.

In summary, MZB1 stands at the forefront of immunoregulatory research, bridging fundamental immunology and clinical medicine. A balanced and comprehensive exploration of its diverse functions and mechanisms will not only enhance our understanding of immune regulation but also pave the way for innovative approaches to combat tumors and autoimmune diseases. As research progresses, harnessing the full potential of MZB1 promises to yield significant advancements in personalized medicine and improve patient outcomes across a spectrum of immune-mediated conditions.
